# Telehealth—An Environmentally Friendly Way to Take Care of Patients with Inflammatory Bowel Disease

**DOI:** 10.3390/medicina61020332

**Published:** 2025-02-14

**Authors:** Srdjan Marković, Djordje Kralj, Tamara Knežević Ivanovski, Petar Svorcan

**Affiliations:** 1 Department of Gastroenterology, University Hospital Medical Center Zvezdara, 11000 Belgrade, Serbia; drkraljdjordje@gmail.com (D.K.); tamara6788@gmail.com (T.K.I.); svorcanp@mts.rs (P.S.); 2 Faculty of Medicine, University of Belgrade, 11080 Belgrade, Serbia

**Keywords:** telehealth, eco-friendly, digital health, telemedicine, inflammatory bowel disease

## Abstract

*Background and Objectives:* On 11 March 2020, our hospital adapted to the COVID-19 pandemic by becoming a temporary COVID-19 facility, leading to the suspension or delegation of non-COVID-19 services. Among the international IBD community, there were significant concerns regarding the neglect of immunocompromised IBD patients and their increased vulnerability to COVID-19. To address these challenges, the COVID-19 ECCO Taskforce recommended the implementation of telehealth. Following this recommendation, our hospital’s IT department integrated audiovisual hardware and software solutions to facilitate virtual consultations. This approach enabled patients and their local physicians to receive formal reports comparable to those issued during standard in-person care. *Materials and Methods:* We retrospectively analyzed data from patients diagnosed with Crohn’s disease and ulcerative colitis who participated in telemedicine consultations. Average distances and time saved were calculated using Google Maps, while carbon emissions and carbon footprint reductions were determined. *Results:* Between 11 August 2021 and 15 June 2023, 107 telehealth consultations were completed. Patients benefited from reduced travel distances, with an average saving of 168.28 km per consultation and a total reduction of 18,006 km. Travel time savings averaged 2 h and 22 min per consultation, amounting to a total of 252 h saved. The reduction in carbon emissions was calculated at 3.26 tons, equivalent to the annual absorption capacity of 109 fully grown trees, considering that an individual tree absorbs approximately 21.77 kg of CO_2_ annually. These findings underscore telemedicine’s role in reducing environmental impact while enhancing patient convenience. *Conclusions:* The adoption of telehealth successfully optimized outpatient clinic operations, maintaining high-quality patient outcomes while contributing to environmental sustainability.

## 1. Introduction

Inflammatory bowel disease (IBD), encompassing both Crohn’s disease and ulcerative colitis, represents a significant global health challenge with a substantial socioeconomic impact. The prevalence of IBD is rising steadily, with over one million individuals affected in the United States alone, and other regions of the world experiencing similar trends. Currently, it is estimated that over one million individuals in the United States and 2.5 million in Europe are affected by IBD, contributing to substantial healthcare costs [1]. These chronic illnesses demand continuous management, due to their unpredictable nature of flare-ups and remissions. Effective management often necessitates regular consultations, putting strain on both healthcare systems and patients.

The COVID-19 pandemic further complicated access to healthcare, as many hospitals, including our own University Hospital Medical Center “Zvezdara”, redirected resources to manage COVID-19 cases. Subsequently, non-essential services, particularly those concerning chronic conditions like IBD, faced suspension. This led to the rapid adoption of telehealth technologies, guided by recommendations from health organizations and the COVID-19 ECCO Taskforce, with the aim of protecting our immunocompromised patients and providing continuous healthcare access. This shift not only addressed immediate healthcare needs, but also opened avenues for integrating environmental sustainability into patient care by minimizing travel.

IBD is a chronic condition characterized by alternating periods of active disease (flares) and remission [2]. As a result, patients with IBD require consistent access to their healthcare providers. The recurrent nature of the disease imposes a significant economic and health burden on patients, their families, healthcare systems, and nations [3]. Notably, only 40% of patients adhere adequately to their therapeutic regimens. Nonadherence to medical therapy leads to a fivefold increase in the risk of disease exacerbation. This high rate of nonadherence is often attributed to limited access to specialized IBD consultations, insufficient patient knowledge about IBD, and the critical role of medical treatment in preventing relapses [4]. Studies emphasize that stringent control of disease activity and early intervention during flare-ups are essential to reduce their duration and avoid complications [5]. Furthermore, many biologic therapies come with severe side effects, making personalized and continuous monitoring crucial for these patients [6]. Access to gastroenterologists with expertise in IBD is often limited, particularly for patients with complex conditions who live in rural areas, who must travel significant distances for specialized care [1].

A substantial portion of patients require brief consultations, especially regarding the initiation of biologic therapies and their impact on everyday life. These immunocompromised individuals are at an increased risk of severe infections while waiting in crowded consultation rooms. The demands of modern life call for innovative solutions, pushing the healthcare system to adapt to the fast-paced and stressful lifestyles of the 21st century. Telemedicine and telehealth emerge as potential tools to enhance monitoring and improve access to specialized IBD care.

Telemedicine involves electronic communication between patients and healthcare providers, or among providers themselves, to optimize patient care. This includes methods such as text messaging, email, video conferencing, patient portals, and remote monitoring programs. Numerous studies have assessed the feasibility, patient satisfaction, effectiveness, healthcare utilization, and educational benefits of telemedicine systems in the context of IBD [7].

Diagnosing IBD is challenging, and requires both endoscopic and radiologic evaluations [2]. During the diagnostic process, telemedicine can help to assess symptoms and review pathological findings through telepathology. Additionally, it facilitates remote monitoring of symptoms and enables the collection of diagnostic data, such as body weight and fecal calprotectin levels, via home point-of-care testing [7]. Telemedicine is crucial in managing IBD, as it allows for close monitoring of medical treatments, supports adherence, enhances communication between patients and physicians, and enables collaboration within multidisciplinary medical–surgical teams [8].

In our clinical practice, we offer telehealth services as an alternative to an office visit for follow-up after initial consultation.

On 11 March 2020, our medical practice underwent a significant transformation due to the COVID-19 pandemic. Our hospital, like many others, was repurposed into a temporary COVID-19 treatment facility, necessitating the suspension or redirection of all non-COVID-19-related medical services. Recognizing the heightened vulnerability of our immunocompromised IBD patients, we swiftly adopted telehealth solutions, as recommended by the COVID-19 ECCO Taskforce.

At our Gastroenterology Department at University Hospital “Zvezdara”, located in Belgrade, Serbia, which is a tertiary referral center for IBD, we have embraced telemedicine, especially since the outbreak of the SARS-CoV2 pandemic. Since then, we have started to use telemedicine in our daily practice with IBD outpatients. At first, throughout phone consultations and text messages, we provided timely care during the pandemic and helped patients to reduce unnecessary doctors’ visits.

The indications and clinical situations for telemedicine usage in IBD patients are presented in Table 1.

Our IT department efficiently integrated audiovisual hardware and software, enabling us to conduct patient consultations via video links. These virtual consultations concluded with formal documentation sent to both patients and their local physicians, maintaining the standard of care provided during in-person visits.

The purpose of this manuscript is to evaluate patient experiences with telemedicine and present our center’s observations and findings.

## 2. Materials and Methods

We retrospectively enrolled patients diagnosed with Crohn’s disease and ulcerative colitis who participated in telemedicine consultations.

In our retrospective study, we analyzed telemedicine consultations conducted for patients with IBD from August 2021 to June 2023. Using Google Maps, we calculated the average travel distance between the hospital and their home address, and the time saved per consultation. To estimate the reduction in carbon emissions, we utilized tools such as CarbonFootprint.com and CO2Meter.com.

The patients studied included those with stable IBD conditions, allowing telehealth to efficiently manage their periodic needs for medication assessments, lab result interpretations, and necessary scheduling for in-person procedures like colonoscopies, thus optimizing in-person visits only when indispensable. Moreover, a financial analysis considered savings from fuel costs, decreased vehicle maintenance, and reduced need for accommodation and work absences. We conducted an analysis of costs, focusing on travel-related expenses. Based on the total distance saved, which was calculated as 18,006 km, we estimated fuel savings, assuming an average fuel consumption of 7 L per 100 km and a fuel cost of 1.5 EUR per liter. Further cost-saving measures included reductions in indirect expenses, such as vehicle wear and tear, missed workdays, and overnight stays.

## 3. Results

From 11 August 2021 to 15 June 2023, we conducted 107 telehealth consultations. The 107 teleconsultations were related to 90 unique patients, indicating repeated sessions with some individuals for continuous monitoring and assessment. Approximately 50% of these teleconsultations prompted the necessity for more decisive action, such as scheduling a face-to-face assessment and diagnostic examination. This decision was determined by a combination of symptom severity, the need for physical examination, and the limitations of remote diagnosis. Following the teleconsultation, almost all of the consultations led to adjustments in the patient’s treatment plan. This included changes in medication, changes in dosage, or the introduction of new management strategies. Regarding return consultations for inflammatory diseases, almost 100% of the consultations were designated as follow-up sessions, of which about 50% were teleconsultations. These were scheduled based on the routine care and monitoring requirements for managing such conditions effectively.

The average distance saved per patient was 168.28 km, totaling 18,006 km saved. This translated to an average travel time saving of 2 h and 22 min per consultation, amounting to a total of 252 h saved. The reduction in carbon footprint was 326 tonnes, which is significant considering that the annual average carbon footprint is 4.65 tones per person in Serbia. This reduction is equivalent to the annual carbon absorption of 109 fully grown trees.

Beyond fuel costs, additional savings were identified in areas such as reduced vehicle wear and tear, decreased time off work, and the elimination of overnight stays. Patients often incur expenses for overnight stays when traveling to major cities, such as Belgrade, for specialized consultations. By eliminating the need for such travel, telemedicine enabled an estimated saving of 1500.50 EUR in accommodation costs, assuming an average cost of 50 EUR per night. Furthermore, patients saved valuable workdays, which, at an average daily wage of 30 EUR, translated into an additional saving of 1800.60 EUR.

In total, the use of telemedicine generated a combined saving of 5191.73 EUR for patients. A summary of our results is shown in Table 2.

## 4. Discussion

The study demonstrated that telehealth services significantly decreased travel requirements for patients. Our findings highlight not only the economic benefits of telemedicine, but also its potential to alleviate the logistical and financial burdens faced by patients, thereby improving access to care and overall patient satisfaction. Such advantages underscore the importance of telemedicine as a sustainable and patient-centered approach to managing chronic conditions like IBD.

Telemedicine has transformed the management of chronic conditions, such as inflammatory bowel disease (IBD), by offering continuous care to patients, alleviating the strain on healthcare systems, and delivering significant environmental and economic advantages. Our findings underscore that telemedicine not only sustains the quality of care for IBD patients, but also enhances patient convenience and promotes sustainability. The adoption of telehealth during the COVID-19 pandemic nudged us towards innovative healthcare delivery models that were previously underutilized. Compared to global studies, our results reflect similar environmental and economic benefits. For instance, studies from other regions like Canada and the UK substantiate the cost reductions and eco-friendly outcomes of telemedicine, complementing our findings.

Telehealth also demonstrates advantages in healthcare resource optimization, serving as an intervention for communities with limited access to specialized care due to geographic or economic barriers. A shift toward remote patient monitoring and virtual consultations not only decreases the healthcare system load, but it also encourages proactive patient involvement in self-management strategies via mobile apps and online platforms.

Despite its benefits, telehealth implementation is not without challenges. Issues such as technological accessibility, data security, and coverage of telehealth services under insurance policies need careful consideration and resolution. Moreover, the need for developed infrastructure, including high-speed internet, becomes apparent in facilitating such advancements.

The accelerated adoption of telehealth during the COVID-19 pandemic highlighted its potential to optimize outpatient services, reduce reliance on in-person visits, and lower the risks associated with travel and exposure to infections. Our study revealed considerable reductions in travel time and expenses, accompanied by a notable decrease in carbon emissions, showcasing the dual benefits for patients and the environment.

Remote monitoring has been shown to be an essential tool for managing IBD in outpatient settings. Various remote methods, including disease activity monitoring, telehealth visits, teleconsultations, and home-based monitoring, are widely used to assist clinicians in managing patients [7]. Remote monitoring of disease activity involves evaluating symptoms, collecting diagnostic data such as body weight, and at-home testing for biomarkers like fecal calprotectin [7]. Several applications have been developed to monitor patients’ conditions, assess their quality of life, and provide insights into disease activity.

One prominent example is the HealthPROMISE platform, a unique cloud-based patient-reported outcomes (PRO) and remote patient monitoring (RPM) system developed by the AppLab at the Icahn School of Medicine at Mount Sinai. This tool enables patients to monitor their quality of life (QOL) and symptoms, while allowing providers to view real-time data. HealthPROMISE aids healthcare providers in addressing gaps in care and informing treatment decisions that enhance QOL and clinical outcomes. Remarkably, 93% of users recommended this platform to other IBD patients [9].

De Jong et al. introduced the myIBDcoach telemedicine system to assist patients and providers in the Netherlands with daily IBD management. This platform supports patients in monitoring disease activity, tracking QOL, and securely communicating with care teams. In a feasibility study, patients rated myIBDcoach at 7.8 out of 10, while providers gave it a rating of 8.0 for design and accessibility. After 12 months, the telemedicine group exhibited fewer outpatient visits to gastroenterologists or nurses (1.55 vs. 2.34, *p* < 0.0001) and fewer hospital admissions (0.05 vs. 0.10, *p* = 0.046). The study concluded that telemedicine is safe and offers significant potential for restructuring IBD care toward personalized, value-based models [10].

The GI Buddy app, developed in 2012 by the Crohn’s and Colitis Foundation of America, was designed to promote self-management and strengthen patient–provider communication. This app helps patients to track their daily symptoms, emotional well-being, medication schedules, and medical histories, while allowing them to prepare questions or notes for clinical visits [11].

Fecal calprotectin, a stool-based biomarker for intestinal inflammation, is widely utilized in disease monitoring due to its non-invasive nature. Ostlund et al. demonstrated that home-based fecal calprotectin monitoring using a digital application was both feasible and well received. Notably, compliance with the IBD-Home model was higher in women, and associated with improved treatment outcomes [12].

Telepathology, which involves electronically transmitting pathology slide images, plays a crucial role in confirming dysplasia in surveillance biopsies from patients with chronic colitis [13]. Furthermore, patients with IBD often encounter difficulties in accessing specialized gastroenterologists, requiring time-intensive and costly travel. Tele-visits using online video conferencing provide a cost-effective alternative to routine office appointments, addressing disparities in access [7].

At Dartmouth–Hitchcock Medical Center, a study involving 48 IBD patients participating in telehealth visits revealed that 81% of patients resided over 25 miles from their providers. Through telehealth, patients reported saving between half a day to an entire day of time, with average cost savings of $62 per visit (ranging from less than $50 to more than $200) [7].

Technological advances have not only enhanced access to care, but also facilitated real-time teleconsultations, enabling live case discussions and multidisciplinary decision-making [14]. Additionally, tele-education initiatives, often delivered through high-quality web portals or online courses, have expanded access to medical education and training, supporting healthcare delivery in remote regions [15].

Our findings align with those of other studies evaluating the economic and environmental benefits of telemedicine, further supporting its utility as a cost-saving and environmentally sustainable approach. A study conducted in Ontario, Canada, demonstrated that telemedicine consultations prevented approximately 3.2 billion kilometers of patient travel, resulting in 545 to 658 million kilograms of carbon dioxide emissions avoided and savings of $569 to $733 million CAD in fuel and transit expenses [16]. Similarly, a study in the United Kingdom estimated that virtual appointments could reduce approximately 232 million road miles annually, leading to significant decreases in carbon emissions and travel-related expenses [17].

These findings are comparable to our results. In addition to financial savings, we observed significant reductions in carbon emissions, highlighting the environmental benefits of telemedicine. Such findings emphasize telemedicine’s role in reducing healthcare’s carbon footprint, which aligns with broader global sustainability goals.

While the economic and environmental benefits of telemedicine are evident, they may vary depending on regional factors such as healthcare infrastructure, population density, and patient demographics. Studies conducted in countries with robust telecommunication networks and centralized healthcare systems may demonstrate more pronounced benefits compared to those with fragmented systems or limited internet access [18]. Furthermore, specific telemedicine modalities, including video consultations and remote monitoring, may yield differing outcomes in cost savings and environmental impact [18].

Telehealth presents transformative potential in managing chronic illnesses like IBD, particularly during global crises that hinder traditional access to medical care. Our study highlights its dual capacity to sustain quality patient care and promote environmental sustainability. By reducing the need for travel-based consultations, telehealth aligns with broader goals of reducing healthcare’s carbon footprint, and offers an economically viable model that is adaptable to other chronic disease management scenarios.

Overall, telemedicine represents a promising solution for addressing healthcare access challenges while simultaneously reducing environmental harm. Future research should focus on optimizing telemedicine systems to maximize both economic and environmental benefits, particularly in underserved and rural populations [18].

The future of telemedicine in IBD management holds significant promise, with several key areas identified for further advancement:

1. Advanced Remote Monitoring Tools: The integration of more sophisticated remote monitoring technologies, including wearable devices and mobile health apps, can enable real-time tracking of patient health metrics. These tools can support early identification of disease flares, allowing timely interventions and further personalization of care.
2. Artificial Intelligence and Machine Learning: The application of AI and machine learning has the potential to enhance predictive analytics, enabling the anticipation of disease exacerbations and optimizing treatment strategies based on individualized data. AI also holds promise for interpreting complex datasets, improving diagnostic precision, and optimizing outcomes.
3. Enhanced Patient Engagement Platforms: Developing more interactive and user-centric telemedicine platforms can improve patient engagement and adherence to therapeutic regimens. Incorporating features such as virtual support groups, educational content, and personalized health coaching can empower patients to take greater ownership of their care.
4. Integration with Electronic Health Records (EHRs): Seamless integration of telemedicine systems with EHRs can improve care coordination, providing healthcare professionals with comprehensive access to patient histories and fostering more effective collaboration across specialties.
5. Tele-education and Training: Expanding virtual education programs for both patients and healthcare providers can enhance disease understanding and management. Continuous professional education through webinars and virtual training can ensure that providers remain informed about the latest developments in IBD care.
6. Policy and Reimbursement Frameworks: Establishing supportive policies and reimbursement structures for telemedicine will be critical for its sustainability and accessibility. Addressing regulatory challenges and promoting equitable access, particularly in underserved and rural areas, is essential for broader adoption.

## 5. Conclusions

Looking forward, it is pertinent to focus on refining telemedicine infrastructure for broader accessibility and integrating advanced digital tools to ensure comprehensive healthcare delivery. Effective policy frameworks and financial incentives can further accelerate telehealth adoption, ensuring its beneficial impact both environmentally and economically. As we advance, embracing such innovations can solidify telehealth’s place as a cornerstone of 21st-century healthcare, ensuring patient-centered and environmentally responsible care models.

In conclusion, telemedicine has already achieved substantial progress in enhancing the management of IBD. However, opportunities for further innovation and refinement remain abundant. By embracing advanced technologies and prioritizing patient-centered care, telemedicine can continue to evolve, offering even more efficient, effective, and personalized solutions for IBD patients worldwide.

## Figures and Tables

**Table 1:** Clinical situations where telemedicine should be used for IBD patients.

Therapy safety and efficacy—short-term follow-up (i.e., mesalamine, azathioprine, biologics)
Follow-up of patients in remission—evaluating laboratory results (i.e., fecal calprotectin…)
Scheduling necessary patient examination, i.e., colonoscopy, MRI, CT scan…
Evaluating histopathology findings after performing endoscopic examination
Patient follow-up after therapy cessation
Evaluation of documentation, which is needed for introduction of biologics

**Table 2:** Summary of our results.

Total Number of Telehealth Consultations	107
Average distance saved per consultation (km)	168.28
Total distance saved (km)	18,006
Average travel time saved per consultation (hours and minutes)	2 h 22 min
Total travel time saved (hours)	252
Carbon footprint reduction (tones)	3.26
Equivalent to annual carbon absorption of fully grown trees	109
Fuel savings (liters)	1260
Fuel cost savings (EUR)	1890.63
Accommodation cost savings (EUR)	1500.5
Workday savings (EUR)	1800.6
Total cost savings (EUR)	5191.73

## Data Availability

The original contributions presented in this study are included in the article. Further inquiries can be directed to the corresponding author.
